# Towards a more precise definition of postoperative pancreatic fistula (POPF): should drain position be considered alongside drain amylase levels after pancreaticoduodenectomy? – a retrospective cohort study

**DOI:** 10.1007/s00423-025-03876-5

**Published:** 2025-10-08

**Authors:** Mohamed Wael, Mohamed I. Kassem, Hashem Altabbaa, Mostafa Ibrahim Ahmed Seif-Eldeen, Mostafa Refaie Elkeleny, Islam El-Sayes

**Affiliations:** 1 Alexandria Main University Hospital, Alexandria, Egypt; 2 Faculty of Medicine, Alexandria University, Alexandria, Egypt; 3 The Research Papyrus Lab, Alexandria, Egypt

**Keywords:** Pancreatic fistula, PD, Clinically relevant postoperative pancreatic fistula, ISGPS, Surgical drains

## Abstract

**Background:**

The International Study Group of Pancreatic Fistula (ISGPF) defines postoperative pancreatic fistula (POPF) based on drain fluid amylase (DFA) levels. However, drain malposition can compromise the reliability of DFA in detecting POPF. This may trigger premature drain removal, delayed recognition of clinically relevant POPF (CR-POPF), and escalation of morbidity. We evaluated how drain position modifies the diagnostic performance of DFA after PD.

**Method:**

A retrospective analysis of 334 patients who underwent PD (June 2019-December 2023). Drain position was assessed using postoperative imaging and DFA levels were measured on postoperative day3. The primary aim was the incidence of drain malposition, while secondary aims included its impact on the diagnosis of CR-POPF, SFC.

**Result:**

Among 334 patients, the drain was well-positioned in 255 patients (76.4%) and malpositioned in 79 patients (23.6%). High DFA levels were strongly associated with proper drain positioning (152/164, 92.7%). However, low/normal DFA levels often reflected drain malposition, with 67/170 patients(39.4%) incorrectly classified, leading to potential false-negative results. Malpositioned drains patients had a significantly higher SFC(*p* < 0.001) and longer hospital stay (*p* < 0.001).

Multivariable analysis confirmed drain malposition as an independent predictor of CR-POPF (OR 4.74, 95% CI: 1.86–12.06, *p* = 0.001), small pancreatic duct(OR 10.59, 95% CI: 3.92–28.62,*p* < 0.001) and low preoperative albumin (OR 0.07, 95% CI: 0.02–0.24, *p* < 0.001).

**Conclusion:**

Drain malposition significantly impacts DFA reliability with false-negative diagnosis. Revising the ISGPS definition to incorporate drain positioning alongside DFA levels may improve diagnostic accuracy in defining POPF.

## Introduction

Pancreaticoduodenectomy (PD) is a technically demanding surgical procedure, frequently performed for various benign and malignant pancreatic pathologies. Despite advancements in surgical techniques and perioperative management, postoperative pancreatic fistula (POPF) remains a major complication associated with significant morbidity, including extended hospital stays, postoperative bleeding, wound issues, and ultimately sepsis and increased mortality if not recognized and managed promptly [1, 2].

Until 2005, numerous studies have been conducted on POPF resulting in significantly varied outcomes due to the diverse definitions of POPF utilized in each study [3–5]. To standardize the POPF definition, the International Study Group of Pancreatic Fistula (ISGPF) introduced a widely accepted criteria in 2005, later revised in 2016. According to the ISGPF, POPF is diagnosed when DFA levels exceed three times the upper limit of serum amylase on or after postoperative day 3 (POD3). This biochemical criterion has been instrumental in classifying POPF into biochemical leaks (previously grade A) and CR-POPF, grades B and C). [6, 7]

However, an important limitation of this definition is that it assumes surgical drains remain optimally positioned near the pancreatic anastomosis intraoperative and throughout the entire postoperative period to ensure the pancreatic collection drainage if unfortunately present. Studies suggest that intra-abdominal drains may dislocate in up to 30% of cases, shifting away from the pancreatic anastomosis and leading to unreliable DFA measurements [8, 9].

Drain placement following PD is performed with the dual objective of monitoring pancreatic anastomotic integrity and facilitating early drainage in the event of leakage. However, drain malposition following PD can result in false-negative DFA readings, leading to an underestimation of POPF and potentially delaying intervention. Additionally, misplaced drains may fail to adequately evacuate accumulating fluid, increasing the risk of significant fluid collections (SFC), secondary infections, and sepsis.

We hypothesized that malposition of the drain adjacent to the pancreaticoenteric anastomosis leads to false-negative DFA results and subsequent premature drain removal, delayed interventions, and higher CR-POPF and morbidity. In other words, relying solely on DFA levels for POPF diagnosis is insufficient unless drain position is simultaneously confirmed. Therefore, this study aims to determine the prevalence of drain displacement after PD and consequently evaluating the influence of drain malposition on DFA reliability and POPF diagnosis. By incorporating postoperative imaging-based drain position assessment, our study challenges the current ISGPF definition of POPF and proposes an updated diagnostic approach that integrates both drain placement and DFA levels for improved clinical decision-making.

## Materials and methods

### Study design

This was a retrospective cohort study conducted at Alexandria university hospital evaluating patients who underwent PD between June 2019 and December 2023. The study was approved by the Alexandria University Ethical Committee on Human Research (IRB Number: 00012098), and the requirement for informed consent was waived due to the retrospective nature of the study.

### Patient selection

A total of 376 patients were initially screened for inclusion

#### Inclusion criteria

A) Patients who underwent elective PD for benign or malignant indications. B) Availability of DFA measurements on postoperative day 3 (POD3). C) Postoperative computed tomography (CT) imaging confirming drain position.

#### Exclusion criteria

A) Patients in whom PD was not completed due to intraoperative complications. B) Absence of POD3 DFA measurements or unreported values. C) Cases where drains were accidentally removed or slipped before CT imaging. D) CT abdomen data was missing from our records or CT was not performed during the postoperative period. Patients with missing POD3 DFA values or missing postoperative CT imaging were excluded from the study. Consequently, no statistical imputation for missing data was required.

**Fig. 1:**
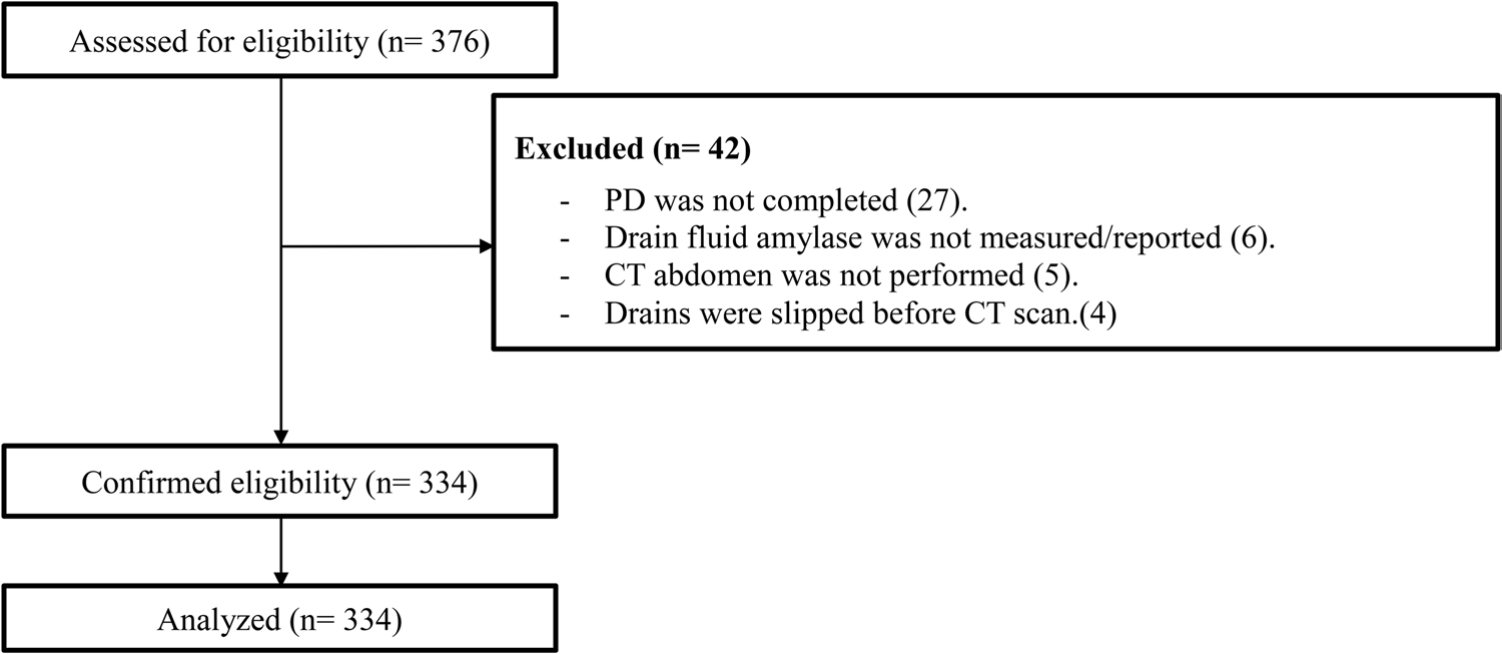
Flow chart showing the included and excluded patients in the study

After applying the exclusion criteria, 334 patients were eligible for analysis (Fig. 1).

### Data collection

The following perioperative and postoperative variables were extracted from medical records. a) Demographic & Preoperative Data including age, gender, and body mass index (BMI), general health status of the patients according to the American Society of Anesthesiologists classification and primary Indications for undergoing PD b) Intra-operative variables such as pancreatic texture (soft vs. firm), pancreatic duct diameter (mm), type of pancreaticoenteric anastomosis, and duration of the operation (minutes) c) 3. Postoperative Variables: DFA levels on POD3, drain position (confirmed via CT imaging), clinically relevant POPF (CR-POPF) development, significant fluid collections (SFC), 30-day postoperative complications, reoperations, and mortality.

### Study definitions

- **Performance status (PS)** was assessed using the ECOG PS scale [10].
- **Postoperative pancreatic fistula (POPF):** POPF was defined according to the 2016 International Study Group of Pancreatic Surgery (ISGPS) criteria [11]
  a) Biochemical leak (categorized previously by previous versions of the ISGPS as grade A POPF) was any measurable drain fluid volume starting from POD 3, with an amylase drain level more than triple the upper normal amylase limit in our institute, with still no clinical affection; b) Grade B POPF: persistent amylase rich fluid drainage over three weeks or those experienced specific interventions such as percutaneous drainage. C) Grade C POPF: Organ failure, reoperation, or mortality directly related to POPF. CR-POPF collectively includes only Grade B and C POPF.[1, 11, 12]
- **Drain position** was defined in the same method as was described by Marchegiani, Ramera, [8] in their study which aimed to assess the actual rate of intra-abdominal drains dislocation. Drain position was evaluated via postoperative CT imaging and categorized as mal-positioned if is tip was located away from the nearby anastomosis, either in a different abdominal region in the coronal plane, or in a significantly more ventral position than the standard position in the axial plane.
- **Significant fluid collections (SFC)** was defined as postoperative intra-abdominal fluid collections associated with clinical symptoms (abdominal pain, fever), leukocytosis (WBC > 12,000/μL), or elevated CRP requiring antibiotics or specific interventional measures as fluid sampling or drain insertion [13].
- **30-day postoperative outcomes**:
   Postpancreatectomy hemorrhage (PPH) was graded according to the ISPGS consensus definition [14].
  Delayed gastric emptying (DGE): radiographic proof of delayed gastric emptying or the need for nasogastric tube 127 decompression for more than two weeks after surgery [15]. Reoperation refers to any surgical intervention within 30 128 days of PD. Operative mortality was defined as in-hospital death or death within 30 days of surgery.

### Surgical technique and drain management

#### Surgical technique

All procedures were performed by the same surgical team following a standardized PD approach with either pylorus-preserving or resecting techniques. Continuity was re-established by triple anastomosis; pancreaticoenteric, gastroenteric and bilioenteric anastomosis. Pancreaticojejunostomy was the usually preferred pancreaticoenteric anastomosis in our institute.

#### Drain placement

**Fig. 2:**
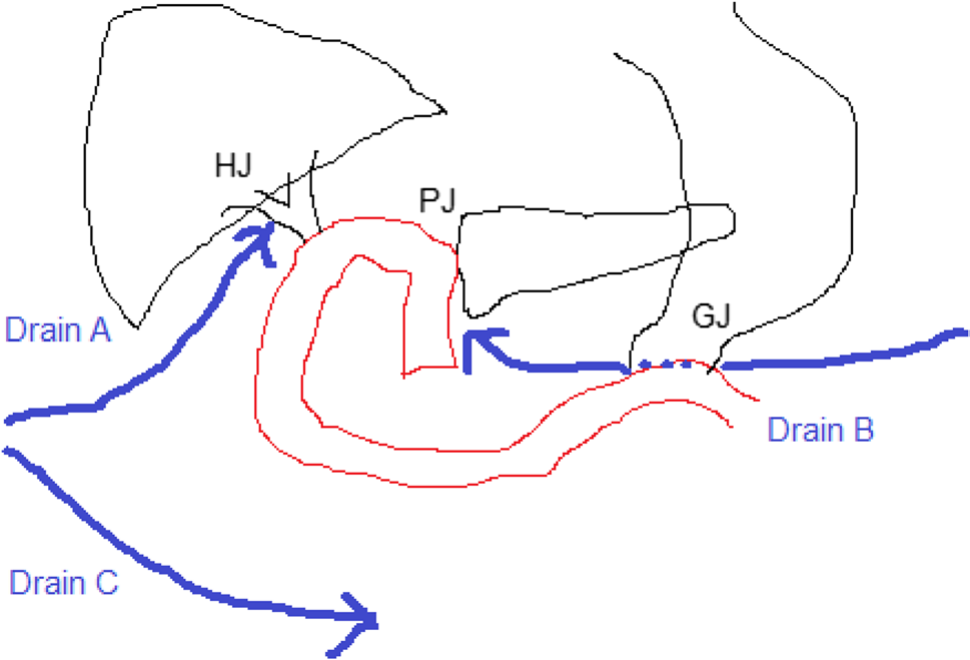
Intraoperative placement of drains according to our institution policy. HJ; hepaticojujonostomy, PJ; pancreaticojujonostomy, GJ; gastrojujonostomy

Non-suction passive tube drains (silicone tube drains, 18Fr) were placed, in all patients, in the surgical field through separate skin incisions. According to our institution, drains after PD were usually positioned as follows (Fig. 2); Drain "A": Posterior to the biliodigestive anastomosis, Drain "B": Adjacent to the pancreaticojejunal anastomosis, Drain "C": Positioned in the pelvic cavity. Drain "B": The drain adjacent to the pancreaticojejunal anastomosis was considered the critical drain for DFA assessment.

### Postoperative care and drain management is standardized for PD patients in our institution

Patients remained nil-by-mouth with nasogastric tube decompression. If the Ryle's tube output remains below 100 ml, the Ryle's tube was removed on POD2. Feeding was then gradually increased based on the patient's tolerance. Typically, patients were able to consume a full diet by POD4. On POD3; DFA levels were routinely tested and a low-dose, non-enhanced computed tomography (LDCT) study was performed. Drains were subsequently removed in case of non-suspicious output, drain amylase level was non-significant (according to the ISGPS definition of pancreatic fistula) or a LDCT revealed absence of a nearby intra-abdominal collection to the drain tip. Our policy correlates with the principles of the ERAS protocol that is widely accepted for postoperative management of PD patients. [16]

### Study endpoints

The primary endpoint of this study was determining the incidence of drain displacement after PD. The secondary endpoints included; a) Evaluation of the impact of drain dislocation on DFA reliability after PD, b) Correlation between drain position and incidence of CR-POPF, and c) Evaluation of overall postoperative morbidity and mortality in relation to drain position.

### Statistical analysis

IBM SPSS Statistics for Windows, version 25.0, was used to perform different statistical analysis. Categorical variables were compared with the chi-square test or Fisher’s exact test. Univariate analysis was performed to identify possible risk factors, and logistic regression was performed to determine independent risk factors. *P*-value of ≤ 0.05 was regarded as significant. Given the exploratory nature of the analyses, we did not adjust for multiple comparisons; this increases the risk of type I error and is acknowledged as a limitation.

A post hoc power analysis was performed using G*Power (version 3.1). Based on the observed difference in CR-POPF incidence between patients with well-positioned drains (15/255, 5.9%) and malpositioned drains (17/79, 21.5%), the achieved power was 95.5% at a two-sided α of 0.05, confirming adequate statistical power to detect this effect size.

## Results

### Patient characteristics

A total of 334 patients who underwent PD between June 2019 and December 2023 were included in the final analysis. The mean age was 55.37 ± 9.12 years, with 59% male and 41% female. The mean BMI was 32.37 ± 2.95 kg/m^2^, and 62% of patients were classified as ASA I (Table 1). Obstructive jaundice was the main presentation in the included patients. Ductal adenocarcinoma (130/334, 39%) was the most common pathology. Pathological diagnosis of included patients is shown in Table 2.

**Table 1:** General characteristics of the included patients

	No. (%)
Age (years)^a^	55.37 ± 9.12
Sex	
Male	198 (59)
Female	136 (41)
BMI (kg/m^2^)^a^	32.37 ± 2.95
ASA Classification	
I	207/334 (62%)
II	98/334 (29%)
III	29/334 (9%)

All values presented in Number (percentage).

^a^mean ± SD

**Table 2:** Patients pathologic diagnosis (*N* = 334)

	No. (%)
Duct adenocarcinoma	130/334 (39%)
IPMN	41/334 (12%)
Mucinous pancreatic neoplasms	16/334 (5%)
Neuroendocrine tumors	37/334 (11%)
Others	110/334 (33%)

All values presented in Number (percentage)

### Drain position and DFA levels

#### Incidence of drain malposition

Postoperative drain positioning plays a crucial role in the accurate interpretation of DFA levels, which serve as a primary diagnostic marker for postoperative pancreatic fistula (POPF). In this study, drain "B" (critical for DFA assessment) was correctly positioned in 255/334 patients (76.4%) but malpositioned in 79/334 patients (23.6%).

#### Correlation between drain position and DFA Levels

A) Among patients with high DFA levels (> 3 × serum reference range, *n* = 164), the vast majority (92.7%, *n *= 152/164) had well-positioned drains, while 12/164 patients only (7.3%) had malpositioned drains. B) Among patients with low/normal DFA levels (< 3 × serum reference range, *n* = 170), 103/170 patients (60.6%) had well-positioned drains, while 67/170 patients (39.4%) had malpositioned drains. These findings highlight that high DFA was strongly associated with proper drain positioning, whereas low/normal DFA levels included a substantial proportion of patients with malpositioned drains (39.4% in this study), indicating the potential for false-negative DFA readings. C) False-negative rate: In this study, 8.8% of patients with low/normal DFA levels developed CR-POPF, despite DFA being < 3 × reference range).

To further analyze the relationship between drain positioning, POD3 drain amylase (DFA) levels, and the subsequent development of CR-POPF, postoperative CT abdomen findings were correlated with clinical outcomes. This analysis revealed significant differences in CR-POPF incidence and intervention requirements based on drain placement and DFA levels.

- Among patients with high DFA levels, drain "B" was properly positioned in 152 patients (152/164, 92.7%) and mispositioned in 12 patients (12/164, 7.3%).
  Well-Positioned drain group (*n* = 152): Nine patients (9/152, 5.9%) developed signs of sepsis, prompting further radiologic evaluation that confirmed SFC. These patients underwent interventional radiology (IR)-guided aspiration or drain insertion, revealing amylase-rich fluid in 6 patients (Grade B CR-POPF). Thirteen patients (13/152, 8.6%) developed CR-POPF, including 7 patients with prolonged drain output (> 3 weeks) and 6 patients with SFC requiring IR-guided drainage, with high amylase levels in the aspirated fluid.
  Malpositioned drain group (*n* = 12): All 12 patients (100%) developed SFC and required IR-guided drainage. However, only 4 of these patients (4/12, 33.3%) had amylase-rich aspirate, confirming Grade B CR-POPF, while the remaining patients (8/12, 66.7%) had sterile or low-amylase collections, suggesting that high DFA levels in malpositioned drains may not always indicate true pancreatic fistula.
  These findings suggest that while high DFA levels are generally indicative of CR-POPF when drains are correctly positioned, mispositioned drains may yield high DFA values that do not always correlate with clinically significant leaks.
- Patients with Normal/Low POD3 DFA Levels (< 3 × Serum Reference Range), a non-significant DFA level (< 3 × reference range) was measured in 170 patients, of whom 15 patients (15/170, 8.8%) still developed CR-POPF, highlighting the risk of false-negative DFA readings when drains are \malpositioned.

Well-Positioned drain group (*n* = 103/170, 60.6%: (Two patients (1.9%) developed CR-POPF despite normal DFA levels: One patient underwent ultrasound (US)-guided drain insertion for a SFC located away from the drain tip. The other patient developed early postoperative wound infection with amylase-rich discharge through the wound, despite no intra-abdominal collection being detected. These cases indicate that even with well-positioned drains, some pancreatic leaks may be underrepresented if fluid is diverted away from the drain collection site.

Malpositioned drain group (*n* = 67/170, 39.4%): Twenty patients (29.9%) developed SFC, requiring IR-guided drainage. Thirteen of these patients (65%) had amylase-rich fluid, confirming CR-POPF. The remaining 7 patients (35%) had sterile or low-amylase aspirates, suggesting that some pancreatic leaks remained undetected due to initial drain misplacement.

These findings demonstrate that low DFA levels do not reliably exclude CR-POPF if the drain is malpositioned. The fact that 19.4% of patients with mispositioned drains in the normal/low DFA group later developed CR-POPF suggests that a significant number of pancreatic leaks are initially overlooked when relying solely on DFA measurements.

## Limitations of DFA levels in predicting postoperative outcomes (Table 3)

**Table 3:** Perioperative data according to drain fluid amylase level

	High amylase level (> 3x) (*n* = 164)	Normal/low level (< 3x) (*n* = 170)	*P*-value
Drain position (drain "B")			< 0.001
Well	152/164(93%)	103/170 (61%)	
Mal	12/164 (7%)	67/170 (40%)	
Significant fluid collection (SFC)	20/164 (12%)	22/170 (13%)	0.870
CR-POPF	17/164 (10%)	15/170 (9%)	0.711
Gastric leak	0 (0)	1/170 (1%)	1
Postpancreatectomy haemorrhage	5/164 (35)	4/170 (2.4%)	0.747
Bile leak	6/164 (3.7%)	4/170 (2.4%)	0.536
Re-operation	0 (0)	1/170 (0.6%)	1
30 day mortality	2/164 (1.2%)	2/170 (1.2%)	1

All values presented in Number (percentage)

Among the 164 patients with high DFA levels (164/334, 49.1%), 152 patients (152/164, 93%) had well-positioned drains, whereas 67 patients (67/170, 39.4%) with normal/low DFA levels had malpositioned drains, suggesting a high risk of false-negative DFA readings. Despite this, the incidence of SFC was similar between the high and low DFA groups (20/164, 12% vs. 22/170, 13%, *p* = 0.870), and CR-POPF rates were also comparable (17/164, 10% vs. 15/170, 9%, *p* = 0.711). Other complications, including bile leak (*p* = 0.536), postpancreatectomy hemorrhage (*p* = 0.747), and 30-day mortality (1.2% in both groups, *p* = 1.00), showed no significant differences based on DFA levels. These findings suggest that DFA levels alone may be insufficient for assessing postoperative risk, particularly in cases where drains are malpositioned, leading to underestimation of POPF. The lack of significant differences in CR-POPF and SFC rates between DFA groups reinforces the importance of verifying drain placement via postoperative imaging before making clinical decisions based solely on DFA levels.

## Impact of drain malposition on CR-POPF

**Fig. 3:**
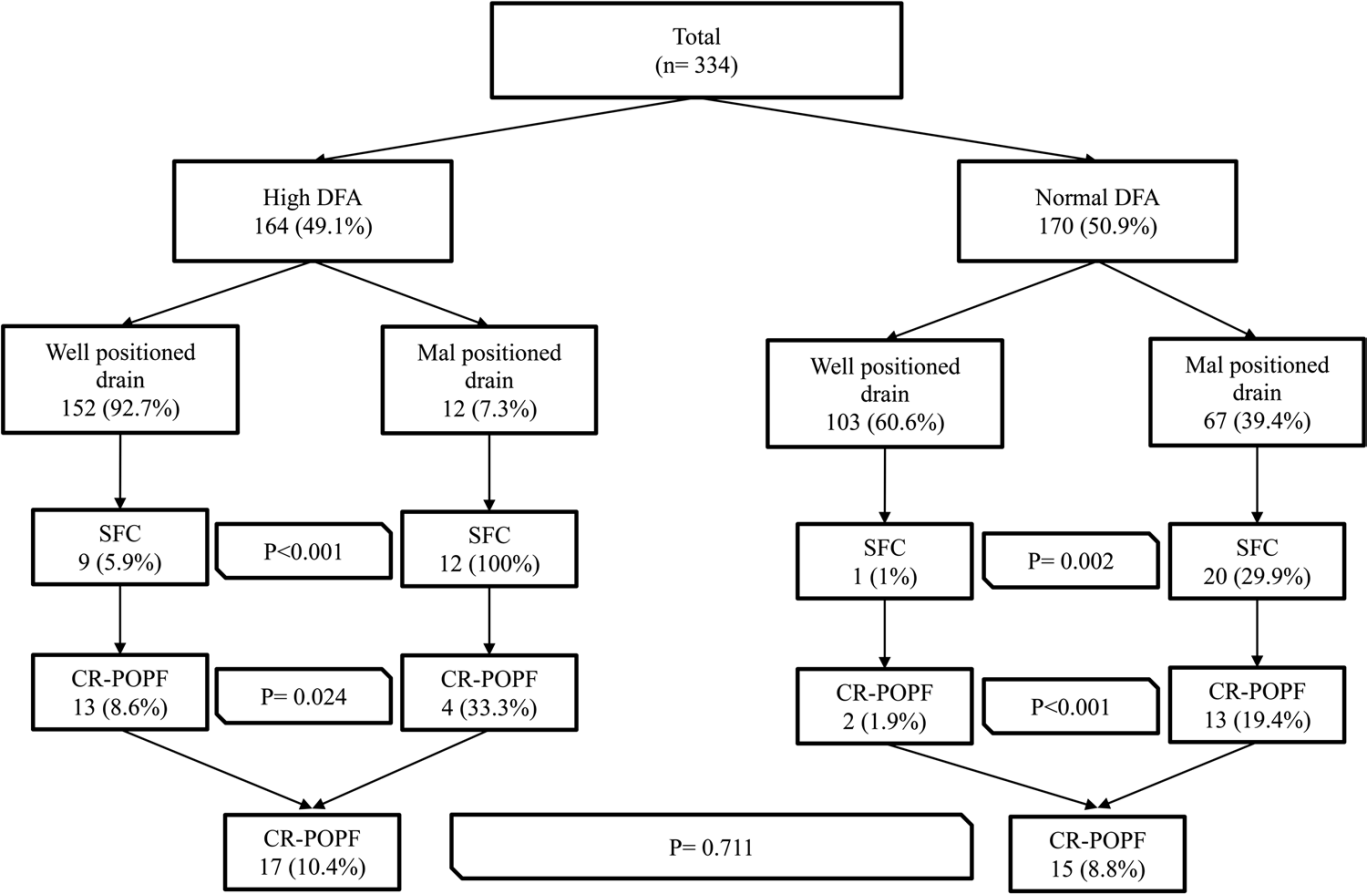
The incidence of CR-POPF according to drain position and DFA levels. DFA; drain fluid analysis, SFC

CR-POPF developed in 32 patients (9.6%) across the entire cohort (Grade B or C). The incidence of development of CR-POPF according to drain position (drain "B") and DFA level is shown in Fig. 3

- Among patients with high DFA levels (*n* = 164), the CR-POPF rate was 17/164 (10.4%). However, there was a substantial difference based on drain position: patients with well-positioned drains (152/164,92.7%) had a CR-POPF incidence of 8.6% (*n* = 13/152), whereas those with malpositioned drains (12/164,7.3%) had a markedly higher incidence of CR-POPF (4/12, 33.3%) (*p* = 0.002). This finding suggests that when DFA is elevated, proper drain positioning allows for early detection and appropriate drainage of pancreatic fluid, whereas malpositioned drains may indicate incomplete drainage or failure to capture accumulating collections, leading to a higher likelihood of clinically significant pancreatic leaks.
- Among patients with normal or low DFA levels (*n* = 170), the overall CR-POPF rate was 8.8% (15/170), but the distribution varied dramatically by drain position. Patients with well-positioned drains (103/170, 60.6%) had an extremely low CR-POPF incidence of CR-POPF (2/103, 1.9%), whereas those with malpositioned drains (67/170, 39.4%) had a significantly higher incidence of CR-POPF (13/67,19.4%), (*p* < 0.001). This discrepancy strongly suggests that a substantial proportion of patients with normal or low DFA levels actually had undetected POPF due to inadequate drain placement, leading to false-negative DFA results. The false-negative rate for CR-POPF in patients with malpositioned drains and low DFA was 19.4%, highlighting a serious flaw in current diagnostic criteria if drain positioning is not considered.

## Impact of drain malposition on significant fluid collections

SFC was also strongly associated with drain positioning. A) Among high DFA patients (*n* = 164), the SFC rate was significantly lower in well-positioned drains (9/152, 5.9%) if compared to malpositioned drain group(12/12,100%) (*p *< 0.001) b) Among normal/low DFA patients (*n* = 170), SFC incidence was lower inpatients with well-positioned drains (1/103, 1%) compared to those with malpositioned drains (20/67, 29.9%) (*p* < 0.001). These findings reinforce the crucial role of proper drain placement in facilitating effective drainage of accumulating pancreatic secretions. Inadequate positioning may not only lead to misdiagnosis but also increase the risk of postoperative fluid collections, infectious complications, and secondary interventions.

## Perioperative variables and clinical Impact of drain malposition (Table 4)

**Table 4:** Comparison of perioperative data in relation to drain "B" position

	Well (*n* = 255)	Mal (*n* = 79)	*P*-value
High DFA	152/255 (60%)	12/79 (15%)	** < 0.001**
SFC	9/255 (3.5%)	33/79 (42%)	** < 0.001**
CR-POPF	15/255 (6%)	17/79 (21.5%)	** < 0.001**
Bile leak	6/255 (2.4%)	4/79 (5%)	0.255
Delayed gastric emptying	0 (0)	1/79 (1.3%)	0.237
Reoperation	1/255 (0.4%)	0 (0)	1
Mortality	3/255 (1.2%)	1/79 (1.3%)	1
Post-operative hospital stay(days)*	8 (7 - 8)	9 (8 - 12)	** < 0.001**
Operative time(min) *	315 (300 - 360)	360 (300 - 420)	**0.01**
Pancreatic texture			0.181
Soft	159/255 (62%)	56/79 (71%)	
Firm	96/255 (38%)	23/79 (29%)	
Transection Method			0.873
Scalpel	202/255 (79%)	64/79 (81%)	
Electrocautery	53/255 (21%)	15/79 (19%)	

All values presented in Number (percentage)

*median (IQR)

Postoperative PD sxcomplications were significantly influenced by drain position, with notable differences observed between patients with well-positioned and malpositioned drains.

- The incidence of significant fluid collections (SFC) was markedly higher in the malpositioned drain group (42.0%) compared to the well-positioned group (3.5%) (*p* < 0.001), underscoring the importance of proper drain placement in preventing undrained fluid accumulation.
- CR-POPF was significantly more common in patients with malpositioned drains (17/79 patients, 21.5%) compared to those with well-positioned drains (15/255 patients, 6.0%) (*p *< 0.001). This reinforces the association between inadequate drainage, unrecognized pancreatic leakage, and the subsequent development of severe complications.
- Operative time was significantly prolonged in patients with malpositioned drains (median 360 min vs. 315 min, *p* = 0.01). This suggests that prolonged surgery may be associated with an increased likelihood of intraoperative drain misplacement or subsequent displacement during the early postoperative period.
- The median length of hospital stay (LOS) was longer in the malpositioned drain group (9 days, IQR: 8–12) compared to the well-positioned group (8 days, IQR: 7–8) (*p* < 0.001), reflecting the burden of increased postoperative morbidity in patients with improper drainage.
- Despite the overall mortality was low (1.3%) and did not significantly differ between groups (*p* = 1.00), the higher 30-day readmission rate in patients with malpositioned drains (14.7% vs. 7.5%, *p* = 0.03) further highlights the potential for unresolved complications and the need for subsequent interventions in this subgroup.

## Risk factors for CR-POPF (Table 5)

**Table 5:** Risk factors for development of CR-POPF

	Univariate analysis			Multivariate analysis		
	No CR-POPF (*n* = 302)	CR-POPF (*n* = 32)	*P*-value	OR	95% CI	*P*-value
Age (years)	56 (50 - 62)	58 (51 −60)	0.619			
Gender (male)	176 (58)	22 (69)	0.344			
BMI (kg/m^2^)	33 (30 - 35)	34 (31.25 - 35)	0.438			
Pancreatic texture (soft)	**187/302 (62%)**	**28/32 (87.5%)**	**0.003**	**3.56**	**1.11** - **11.44**	**0.033**
Drain malposition	**62/302 (20.5%)**	**17/32 (53%)**	** < 0.001**	**4.74**	**1.86** - **12.06**	**0.001**
High DFA	147/302 (49%)	17/32 (53.1%)	0.711			
Transection method (stapler)	241/302 (80%)	25/32 (78%)	0.819			
Preoperative albumin	**3 (2.7** - **3.3)**	**2.4 (2.4** - **2.6)**	** < 0.001**	**0.07**	**0.02** - **0.24**	** < 0.001**
Hemoglobin level	12 (11 - 13)	12 (11 - 12)	0.104			
Blood loss	10 (7 - 15)	15 (7 - 20)	0.141			
Pancreatic duct (< 3 mm)	**61/302 (20%)**	**19/32 (59.4%)**	** < 0.001**	**10.59**	**3.92** - **28.62**	** < 0.001**

All values presented in Number (percentage) or Median (IQR)

Multivariate analysis identified drain malposition, small pancreatic duct diameter, and preoperative hypoalbuminemia as independent risk factors for CR-POPF. Patients with malpositioned drains had nearly five-fold higher risk (OR = 4.74, 95% CI: 1.86–12.06, *p* = 0.001), underscoring the critical role of proper drain placement. A small pancreatic duct diameter (< 3 mm) was the strongest predictor, increasing the risk more than tenfold (OR = 10.59, 95% CI: 3.92–28.62, *p* < 0.001), likely due to the higher susceptibility of a narrow duct to anastomotic leakage. Interestingly, preoperative albumin levels were significantly different between groups. Patients without CR-POPF had a median albumin of 3.0 g/dL (IQR 2.7–3.3), whereas those who developed CR-POPF had markedly lower levels, with a median of 2.4 g/dL (IQR 2.4–2.6) (*p* < 0.001). On multivariable regression, each unit increase in preoperative albumin was strongly protective (OR = 0.07, 95% CI: 0.02–0.24, *p* < 0.001), reducing the risk of CR-POPF by more than 90%.This highlights the importance of nutritional status in anastomotic healing. Other perioperative variables—including age, sex, BMI, operative time, and intraoperative blood loss—were not significantly associated with CR-POPF (all *p* > 0.05), suggesting that technical and nutritional factors play a more decisive role than general demographics.

## Discussion

The placement of drains during abdominal surgeries serves several purposes, including the ongoing removal of extruded secretions, the prevention of potential abdominal infection from retained collections, and the early detection of concerning postoperative issues, such as bleeding or disruption of the surgical connection. [17–19] Postoperative drainage in PD operation offers a distinct additional benefit by allowing for the early detection of POPF. The ISGPS in their definition of POPF depends solely on the level of DFA [7] In 2016, a revised definition was implemented, reclassifying grade A POPF as a biochemical leak without clinical impact. Grade B/C POPF was henceforth defined as CR-POPF. [11, 12]

DFA level has unique advantages in pancreatic surgeries after PD; a) the ISGPS definition of PF depends mainly on drain amylase level on POD3. [11] Drain displacement can result in false negative results, which may mistakenly exclude the diagnosis of POPF and cause a delay in subsequent management. b) Many studies have proposed that elevated DFA level in the early postoperative period is a strong predictor of CR-POPF, although the cutoff and use of amylase levels varies between the different studies.

Randomized trials and meta-analyses have questioned routine drains after PD, particularly in selected, low-risk settings with protocolized early imaging and close monitoring. [20–22] Our findings help reconcile this with the observed harm of malposition: a malpositioned drain can provide false reassurance (spuriously low DFA), delaying diagnosis and treatment of clinically significant collections. In contrast, a no-drain strategy—when coupled with vigilant clinical assessment and early selective imaging—may avoid such false-negative signals. Thus, the issue is not “drain vs no drain” per se, but the diagnostic behavior introduced by a misplaced drain. Our data argue for either accurate placement with verification (and early removal when safe) or vigilance pathways that do not over-rely on DFA in isolation.

Analysis of drain dislocation following abdominal surgery has been the subject of limited research. [8] Existing randomized controlled trials have primarily examined strategies for managing drains, with a focus on policies for their removal postoperatively, assuming that the drains are left in place until they are ultimately removed. However, after surgery, follow-up imaging and/or additional surgeries often showed that the drains placed during the operation had moved, yet there is a lack of dependable published information on the potential outcomes of drain displacement in actual medical practice [9]. Surprisingly, Marchegiani, Ramera [8] conducted a study to evaluate how often surgical drains become displaced following pancreatic surgery. The study found that surgical drains are displaced in about one third of cases, with no variation based on the type of procedure (PD or distal pancreatectomy). Accurate drain placement near the pancreatic anastomosis should be adequate. Otherwise, the surgeon may rely on inaccurate false negative results [9]. This study provides robust evidence that drain malposition significantly affects the diagnosis and management of POPF following PD.

We demonstrated that malpositioned drains were associated with higher rates of false-negative DFA readings, delayed CR-POPF diagnosis, increased incidence of SFC, and a greater need for secondary drainage procedures. Importantly, among patients with normal/low DFA levels, 8.8% still developed CR-POPF, and 19.4% of patients with malpositioned drains in this group had undetected pancreatic leaks, emphasizing the high false-negative rate when drain positioning is not verified. These findings challenge the current ISGPS definition of POPF and highlight the need for incorporating drain positioning into clinical decision-making.

In our study, the correlation between drain positioning, DFA levels, and the accuracy of POPF diagnosis was of interesting significance. In patients with high DFA levels (> 3 × serum reference range, *n* = 164), drain "B" was well-positioned in 152 of 164 cases (92.7%), supporting the assumption that high DFA is a reliable predictor of CR-POPF when the drain is correctly placed. However, among patients with high DFA but malpositioned drains, 12 of 164 patients (7.3%), only 33.3% ultimately had CR-POPF, while others had non-pancreatic collections, suggesting that drain malposition may sometimes lead to overestimation of POPF risk due to amylase contamination from surrounding tissues.

Conversely, low DFA levels (< 3 × serum reference range, *n* = 170) were unreliable in excluding CR-POPF when drains were mispositioned. Among patients with low DFA levels and well-positioned drains (*n* = 103), only only 2 of 103 patients (1.9%) developed CR-POPF, reinforcing that well-placed drains reliably capture pancreatic leaks. By contrast, in patients with low DFA but malpositioned drains (67 patients), 13 of 67 (19.4%) were later diagnosed with CR-POPF, most following the identification of SFC via postoperative CT and subsequent IR-guided drainage, which confirmed amylase-rich aspirates.

These findings emphasize that DFA levels alone are not sufficient to diagnose or exclude CR-POPF without accounting for drain position; a high DFA is only reliable when the drain is well-positioned. This underscores the need for routine postoperative imaging to ensure accurate interpretation of DFA results and prevent delayed or missed diagnoses.

Fluid collections after abdominal surgeries including pancreatic anastomosis is common [23] In our study, malpositioned drains were associated with a dramatically higher incidence of SFC (42.0% vs. 3.5%, *p* < 0.001), reinforcing the role of proper drainage in preventing postoperative fluid accumulation This suggests that drain malposition has a rule in underestimating the incidence of abdominal collections which may be due to POPF after PD with subsequent delay in any needed intervention. However, even in the absence of POPF, drain malposition or malfunction may lead to not adequately draining fluid collections leading to infection, which may alter anastomosis healing eventually leading to POPF.

Biochemical leak (BL) development after PD operation is a common finding but does not complicate the clinical postoperative course. However, BL can eventually progress toward a definite PF. Zironda, Benidetti [24] in their study involving 302 patients indicated that the transition from BL to a definite POPF is common, affecting one-third of BL patients in their research. In our study, drains were mal-positioned in 39.4% of patients with normal DFA making a false under-estimation of the incidence of patients with overall high DFA with subsequent different POPF grades.

Different institutions have different policies regarding drain removal. According to the European ERAS Society, drains should be removed on POD3 if the amylase level is less than 5000 U/L on the POD1. This aligns with our institution's policy of removing drains on POD3 if the amylase level in the drain fluid is not significant. In our research, 170 patients had a normal level of amylase in their drain, leading to the subsequent removal of drains on POD3. Regrettably, 15 of these patients (8.8%) later experienced POPF and needed to have an abdominal drain reinserted through intervention radiology. This indicates that the misplacement of the drain can result in a false negative result for DFA, leading to an incorrect decision for early drain removal. The safety of the early drain removal policy following PD may be questionable if it relies solely on the level of amylase in the drain, as a low level may not rule out the potential development of CR-POPF if relying on a improperly positioned drain to collect amylase from the drain fluid. [9]

Marchegiani, Ramera, [8] in their study, on a total of 91 prospectively enrolled patients who underwent either PD (*n* = 58, 64%) or DP (*n* = 33, 36%) with standard drain placement, concluded that drain dislocation postoperatively is common after pancreatic surgery with no significant difference was found between PD (29%) and distal pancreatectomy (39%) in terms of rate of dislocation (*P* = 0.325). Lee, Yoon, [9] in their study, addressed the impact of drain position on DFA levels, Fluid Collection and POPF after distal pancreatectomy and found that drain malposition was frequent leading to false low incidence of POPF.

Our data support ERAS-aligned, risk-stratified care. [16] Routine CT for all patients may not be cost-effective; however, targeted imaging is justified when (i) POD3 DFA is discordant with clinical findings, (ii) the drain appears malpositioned on early imaging, or (iii) there is unexplained sepsis or rising inflammatory markers. In these scenarios, confirming drain position refines DFA interpretation, potentially averting delayed interventions and unplanned readmissions. The incremental cost and radiation of selective imaging may be offset by preventing complications that prolong length of stay.

Compare to previous literature, our study provides a more detailed analysis of clinical consequences of drain malposition in PD operation by quantifying the rates of false-negative DFA readings, secondary drainage procedures, and CR-POPF severity based on drain position. These findings add novel insight into the mechanisms by which drain misplacement contributes to both over- and underestimation of POPF risk, emphasizing the need to revise current diagnostic guidelines.

This study has several strengths, including its large sample size (*n* = 334), standardized surgical technique, and systematic postoperative imaging evaluation of drain positioning. Additionally, multivariate analysis identified drain malposition as an independent predictor of CR-POPF, alongside small pancreatic duct diameter and preoperative hypoalbuminemia. However, there are some limitations; this is a retrospective study, which may introduce selection bias. Additionally, while we confirmed drain position via postoperative CT, the exact timing of drain displacement (intraoperative vs. early postoperative) remains unclear. A prospective study with serial imaging assessments would provide more precise data on when and why drains become displaced. No formal correction for multiple testing was applied, as the analyses beyond the primary endpoint were considered exploratory. Furthermore, while our study highlights the clinical consequences of drain malposition, future research should evaluate whether specific surgical techniques or fixation methods can reduce the risk of drain displacement.

## Conclusion

This study provides strong evidence that drain malposition significantly affects DFA reliability, increases CR-POPF incidence, and raises the risk of secondary interventions. Our findings suggest that routine postoperative imaging should be incorporated into standard PD management to verify drain positioning before relying on DFA levels for POPF diagnosis. Furthermore, our results challenge the current ISGPS definition of POPF, highlighting the need to integrate both DFA levels and drain positioning into future diagnostic criteria. By addressing the impact of drain malposition, we can improve the accuracy of POPF detection, optimize drain management strategies, and ultimately enhance patient outcomes following PD. Hence, for diagnosing POPF in PD patients based on drain amylase level, it is recommended to ensure proper drain placement for accurate interpretation of POPF.

## Data Availability

No datasets were generated or analysed during the current study.

## Abbreviations

PD: Pancreatoduodenectomy
POPF: Postoperative pancreatic fistula
ISGPF: International Study Group of pancreatic Fistula
POD3: Postoperative day three
CT: Computed tomography
BMI: Body mass index
ASA: American Society of Anesthesiologists classification
PS: Patient performance status
ISGPS: International Study Group of Pancreatic Surgery
DGE: Delayed gastric emptying
PPH: Post-pancreatectomy haemorrhage
CR-POPF: Clinically relevant postoperative POPF
SFC: Significant fluid collections
WBC: White blood cells
CRP: C-reactive protein
LDCT: Low-dose, non-enhanced computed tomography

## References

[1] Pedrazzoli S (2017) Pancreatoduodenectomy (PD) and postoperative pancreatic fistula (POPF): a systematic review and analysis of the POPF-related mortality rate in 60,739 patients retrieved from the English literature published between 1990 and 2015. Medicine (Baltimore) 96(19):e6858. 10.1097/MD.0000000000006858 28489778 10.1097/MD.0000000000006858 PMC5428612

[2] Pulvirenti A, Marchegiani G, Pea A, Allegrini V, Esposito A, Casetti L, Landoni L, Malleo G, Salvia R, Bassi C (2018) Clinical implications of the 2016 international study group on pancreatic surgery definition and grading of postoperative pancreatic fistula on 775 consecutive pancreatic resections. Ann Surg 268(6):1069–1075. 10.1097/SLA.0000000000002362 28678062 10.1097/SLA.0000000000002362

[3] Adamenko O, Ferrari C, Schmidt J (2020) Irrigation and passive drainage of pancreatic stump after distal pancreatectomy in high-risk patients: an innovative approach to reduce pancreatic fistula. Langenbecks Arch Surg 405:1233–1241. 10.1007/s00423-020-02012-9 33084924 10.1007/s00423-020-02012-9 PMC7686191

[4] Wu J, Tang Z, Zhao G, Zang L, Li Z, Zang W, Qu J, Yan Su, Zheng C, Ji G, Zhu L, Zhao Y, Zhang J, Huang H, Hao Y, Fan L, Xu H, Li Y, Yang Li, Song Wu, Zhu J, Zhang W, Li M, Qin X, Liu F (2022) Incidence and risk factors for postoperative pancreatic fistula in 2089 patients treated by radical gastrectomy: a prospective multicenter cohort study in China. Int J Surg 98:106219. 10.1016/j.ijsu.2021.106219 34990829 10.1016/j.ijsu.2021.106219

[5] Halder PJ, Ravindra N (2019) Does the anatomy of the transected pancreatic neck influence post Whipple’s operation pancreatic fistula? Indian J Surg Oncol 10(1):31–36. 10.1007/s13193-018-0747-5 30948868 10.1007/s13193-018-0747-5 PMC6414556

[6] Bassi C, Butturini G, Molinari E, Mascetta G, Salvia R, Falconi M,..., Pederzoli P (2004) Pancreatic fistula rate after pancreatic resection: the importance of definitions. Dig Surg 21(1):54–59. 10.1159/000075943‏ 10.1159/000075943 14707394

[7] Bassi C, Dervenis C, Butturini G, Fingerhut A, Yeo C, Izbicki J, Neoptolemos John, Sarr Michael, Traverso William, Buchler Marcus, International Study Group on Pancreatic Fistula Definition (2005) Postoperative pancreatic fistula: an international study group (ISGPF) definition. Surgery 138(1):8–13. 10.1016/j.surg.2005.05.001 16003309 10.1016/j.surg.2005.05.001

[8] Marchegiani G, Ramera M, Viviani E, Lombardo F, Cybulski A, Chincarini M, Malleo G, Bassi C, Zamboni GA, Salvia R (2019) Dislocation of intra-abdominal drains after pancreatic surgery: results of a prospective observational study. Langenbecks Arch Surg 404:213–222. 10.1007/s00423-019-01760-7 30771076 10.1007/s00423-019-01760-7

[9] Lee JS, Yoon YS, Han HS, Cho JY, Lee HW, Lee B, Yoon Yoo‐Seok, Han Ho‐Seong, Lee Hae‐Won, Jo Yeongsoo, Kang MeeYoung, Lee Eunhye, Park Y (2023) Impact of drain position on drain fluid amylase, fluid collection and postoperative pancreatic fistula after distal pancreatectomy. World J Surg 47(5):1282–1291. 10.1007/s00268-023-06933-6 36763135 10.1007/s00268-023-06933-6

[10] Simcock R, Wright J (2020) Beyond performance status. Clin Oncol 32(9):553–561. 10.1016/j.clon.2020.06.016 10.1016/j.clon.2020.06.016 PMC7365102 32684503

[11] Bassi C, Marchegiani G, Dervenis C, Sarr M, Hilal MA, Adham M, Abu Hilal M, Allen P, Andersson R, Asbun HJ, Besselink MG, Conlon K, Del Chiaro M, Falconi M, Fernandez-Cruz L, del Fernandez- Castillo C, Fingerhut A, Friess H, Gouma DJ, Hackert T, Izbicki J, Lillemoe KD, Neoptolemos JP, Olah A, Schulick R, Shrikhande SV, Takada T, Takaori K, Traverso W, Vollmer C, Wolfgang CL, Yeo CJ, Salvia R, Buchler M (2017) The 2016 update of the international study group (ISGPS) definition and grading of postoperative pancreatic fistula: 11 years after. Surgery 161(3):584–591. 10.1016/j.surg.2016.11.014 28040257 10.1016/j.surg.2016.11.014

[12] Pulvirenti A, Ramera M, Bassi C (2017) Modifications in the international study group for pancreatic surgery (ISGPS) definition of postoperative pancreatic fistula. Transl Gastroenterol Hepatol 2:107. 10.21037/tgh.2017.11.14 29354764 10.21037/tgh.2017.11.14 PMC5763010

[13] Kim JS, Rho SY, Shin DM, Choi M, Kang CM, Lee WJ, Hwang HK (2022) Wrapping the pancreas with a polyglycolic acid sheet before stapling reduces the risk of fluid collection on the pancreatic stump after distal pancreatectomy. Surg Endosc. 10.1007/s00464-021-08387-0 33620565 10.1007/s00464-021-08387-0 PMC8758620

[14] Wente MN, Veit JA, Bassi C, Dervenis C, Fingerhut A, Gouma DJ, Izbicki JR, Neoptolemos JP, Padbury RT, Sarr MG, Yeo CJ, Büchler MW (2007) Postpancreatectomy hemorrhage (PPH)–an international study group of pancreatic surgery (ISGPS) definition. Surgery 142(1):20–25. 10.1016/j.surg.2007.02.001 17629996 10.1016/j.surg.2007.02.001

[15] Panwar R, Pal S (2017) The international study group of pancreatic surgery definition of delayed gastric emptying and the effects of various surgical modifications on the occurrence of delayed gastric emptying after pancreatoduodenectomy. Hepatobiliary Pancreat Dis Int 16(4):353–363. 10.1016/S1499-3872(17)60037-7 28823364 10.1016/S1499-3872(17)60037-7

[16] Lassen K, Coolsen MM, Slim K, Carli F, de Aguilar-Nascimento JE, Schäfer M,..., Dejong CH (2012) Guidelines for perioperative care for pancreaticoduodenectomy: Enhanced Recovery After Surgery (ERAS®) Society recommendations. Clin Nutr 31(6):17–830. 10.1016/j.clnu.2012.08.011‏ 10.1016/j.clnu.2012.08.011 23079762

[17] Mcguire S (1903) Drainage after abdominal section. Trans Annu Session 33:104

[18] Zhang W, Cheng Y, Xia J, Lai M, Cheng N, Liu Z (2018) Prophylactic abdominal drainage for pancreatic surgery. Cochrane Database Syst Rev (6):CD010583. 10.1002/14651858.CD010583.pub4 10.1002/14651858.CD010583.pub4 PMC6513487 29928755

[19] Messager M, Sabbagh C, Denost Q, Regimbeau JM, Laurent C, Rullier E, Sa Cunha A, Mariette C (2015) Is there still a need for prophylactic intra-abdominal drainage in elective major gastro-intestinal surgery? J Visc Surg 152(5):305–313. 10.1016/j.jviscsurg.2015.09.008 26481067 10.1016/j.jviscsurg.2015.09.008

[20] McMillan MT, Malleo G, Bassi C, Allegrini V, Casetti L, Drebin JA, Esposito A, Landoni L, Lee MK, Pulvirenti A, Roses RE, Salvia R, Vollmer CM Jr (2017) Multicenter, prospective trial of selective drain management for pancreatoduodenectomy using risk stratification. Ann Surg 265(6):1209–1218. 10.1097/SLA.0000000000001832 27280502 10.1097/SLA.0000000000001832

[21] McMillan MT, Malleo G, Bassi C, Butturini G, Salvia R, Roses RE, Lee MK, Fraker DL, Drebin JA, Vollmer CM Jr (2015) Drain management after pancreatoduodenectomy: reappraisal of a prospective randomized trial using risk stratification. J Am Coll Surg 221(4):798–809. 10.1016/j.jamcollsurg.2015.07.005 26278037 10.1016/j.jamcollsurg.2015.07.005

[22] Zaghal A, Tamim H, Habib S, Jaafar R, Mukherji D, Khalife M, Mailhac A, Faraj W (2020) Drain or no drain following pancreaticoduodenectomy: the unsolved dilemma. Scand J Surg 109(3):228–237. 10.1177/1457496919840960 30931801 10.1177/1457496919840960

[23] Kleespies A, Albertsmeier M, Obeidat F, Seeliger H, Jauch KW, Bruns CJ (2008) The challenge of pancreatic anastomosis. Langenbecks Arch Surg 393:459–471. 10.1007/s00423-008-0324-4 18379817 10.1007/s00423-008-0324-4

[24] Zironda A, Benedetti A, Calcagno P, Giani A, Mazzola M, Ferrari GC (2023) From biochemical leak to pancreatic fistula after pancreaticoduodenectomy: the role of minimally invasive surgery. HPB 25:S443–S444. 10.1016/j.hpb.2023.07.522

